# Applying Cognitive Stimulation Therapy (CST) on People with Concurrent Visual Impairment and Dementia: A Preliminary Study

**DOI:** 10.3390/brainsci16020168

**Published:** 2026-01-30

**Authors:** Hiu Tung Tsang, Chun Lam Luk, Yee Lam Lo, Armstrong Tat San Chiu, Ben Chi Bun Yip, Winsy Wing Sze Wong

**Affiliations:** 1 Academic Unit of Human Communication, Learning, and Development, Faculty of Education, The University of Hong Kong, Hong Kong SAR, China; thtung@connect.hku.hk (H.T.T.); lclbrian@connect.hku.hk (C.L.L.); 2 Department of Language Science and Technology, Faculty of Humanities, The Hong Kong Polytechnic University, Hong Kong SAR, China; ylam2.lo@connect.polyu.hk; 3 The Hong Kong Society for the Blind, Hong Kong SAR, China; armstrong.chiu@hksb.org.hk; 4 School of Health and Medical Sciences, Tung Wah College, Hong Kong SAR, China; benyip@twc.edu.hk

**Keywords:** cognitive stimulation therapy, dementia, visual impairment, cognitive intervention, functional communication, cognition, language

## Abstract

Background/Objectives: This pilot study explored the applicability and preliminary clinical outcomes of cognitive stimulation therapy (CST), an evidence-based cognitive intervention for people with mild and moderate dementia, in elderly individuals with concurrent dementia and visual impairment. Methods: Seven participants received 14 group CST sessions. Their cognitive and language functions were measured and compared pre-/post-therapy. Results: The treatment adherence was satisfactory. Significant improvements in various cognitive domains and language measures were observed after therapy. Conclusions: The findings suggest that CST can be applied to visually impaired individuals with dementia with seemingly positive outcomes in various cognitive domains. Further studies with a larger sample with an emphasis on multisensory stimulation to facilitate therapy delivery are warranted.

## 1. Introduction

### 1.1. Visual Impairment and Dementia 

Dementia and visual impairment are both prevalent in the elderly population. Globally, the number of people with dementia (PwD) is about 55 million [1] and is projected to reach 152.8 million in 2050 [2]. Dementia, also known as major neurocognitive disorders (NCDs) in the Diagnostic and Statistical Manual of Mental Disorders (DSM-5) [3] (DSM-5; American Psychiatric Association, 2022), is differentiated into different subtypes based on etiologies, such as Alzheimer’s disease, vascular disease, Lewy Body disease, and frontotemporal NCD. The manifestations of neuropsychological symptoms vary among the different subtypes and are associated with their neuropathology. For instance, people with Alzheimer’s disease, characterized by the accumulation of beta-amyloid plaques and neurofibrillary tangles in the brain, experience memory loss of recent events at an early stage. People diagnosed with frontotemporal dementia have early changes in personality and behavior, with language being disproportionately affected in the early stage. Meanwhile, for Lewy Body dementia, visual hallucinations and fluctuations in cognitive performance (especially in attention/alertness) are observed [4]. Visual impairment has recently been confirmed as a risk factor for dementia [5]. At least 2.2 billion of the global population suffer from visual impairment [6]. Its prevalence rises drastically from 1% among adults aged 50–54 to 21% among the elderly aged 85 years or above [7]. In Hong Kong, the prevalence of mild dementia for people aged 70 or above was reported to be around 8.9% [8]. According to the report issued by the HKSAR Census and Statistics Department [9], approximately 165,900 people aged 60 or over were diagnosed with visual impairment. An association between the level of severity of visual impairment and the risk of dementia has been reported in Hong Kong [10]. Therefore, there is a strong need to develop assessments and interventions for people with dementia and visual impairment (PwDVI) that help to improve their functions and quality of life and alleviate caregivers’ burden.

### 1.2. Cognitive Intervention for People with Visual Impairment and Dementia

There is no cure for dementia. The existing pharmacological/non-pharmacological interventions aim at managing/reducing symptoms, slowing progression and improving quality of life. While some medications (e.g., galantamine) may help to slow down cognitive deterioration, side effects and tolerability issues have to be considered with caution [11]. Non-pharmacological cognitive interventions have been extensively developed and utilized for PwD. Various approaches, such as cognitive stimulation, enriched environment, exercise therapy, and computerized cognitive training, have been used to support treatment for PWD, with positive outcomes obtained [12]. Cognitive stimulation therapy (CST), a person-centered group-based cognitive intervention developed by Spector et al. [13], is an evidence-based therapy that integrates both cognitive and social stimulation to encourage implicit learning and provide mental stimulation. It has been recommended by the UK National Institute for Health and Care Excellence (NICE) as an effective group intervention for people with mild to moderate dementia. Recent systematic reviews demonstrated high-quality evidence to support using CST to improve cognitive functions, primarily in global cognition, communication, and social interaction [14,15].

In general, the evidence obtained from CST and other cognitive interventions is primarily based on individuals without significant sensory (i.e., visual and/or hearing) impairments. In most of these studies, participants with visual (or hearing) impairments that hindered participation in assessment and treatment were excluded. Marino et al. [16] reported that more than half of the cognitive intervention studies for older adults excluded participants with hearing and/or visual impairment. In other words, PwDVI has been underrepresented; thus, further studies to identify suitable intervention programs for PwDVI and examine their efficacy are warranted. Given the high-level evidence supporting CST from previous studies and its nature, it is reasonable to explore its feasibility and preliminary efficacy among PwDVI. Firstly, the activity- and discussion-based nature of CST is readily applicable to people with visual impairment. The 14 themed sessions of CST involve multisensory activities, e.g., physical activity (Session 1) and sound (Session 2), which can be implemented for people with visual impairment via minor adjustments to the therapy materials and presentation by CST facilitators during therapy delivery (detailed in Materials and Methods). In addition, the principles of CST, such as multiple sensory stimulation to trigger memory, stimulating language and discussion, being person-centered, and maximizing potential, should work well for people with visual impairment.

### 1.3. Research Objectives

The current pilot study addressed two issues: (1) to study the applicability of conventional CST in PwDVI and (2) to explore the effects of CST in PwDVI in various cognitive domains.

## 2. Materials and Methods

### 2.1. Study Design

The study adopted a case-series approach with multiple measurements taken in the pre-treatment and treatment phases.

### 2.2. Participants

Nine elderly individuals from the Tuen Mun Home for the Aged Blind (a residential home for elderly people with visual impairment) passed screening and were recruited for the current study. The inclusion criteria were: (1) fluent Cantonese speakers (2) having a medical diagnosis of dementia by a gerontologist or suspected to have dementia, with the Montreal Cognitive Assessment 5-Minute Protocol (MoCA-5) [17] score ranging from 2 to 17, which is equal to the Cantonese Mini Mental State Examination (MMSE) [18] score ranging from 10 to 24 by using the conversion table from Wong et al. [19], (3) a diagnosis of mild to moderate visual impairment determined by an optometrist, (4) no other disability including physical illness, learning disability and severe hearing impairment that may affect participation in group activity, (5) not currently on psychiatric medication/cognitive intervention, (6) no history of other neurological diseases, and (7) did not receive any prior CST. The trial was registered at clinicaltrials.gov (ClinicalTrial.gov identifier: NCT06793384 [20]). Ethics for data collection was approved by the Faculty Research Ethics Committee of the Faculty of Education. Further ethical approval was obtained from the Human Research Ethics Committee (HREC) of the University of Hong Kong Faculty of Education (EA240610) for dataset processing and analysis. Written consent was sought from both the participants and their caregivers. Two participants dropped out in the course of the intervention due to health reasons or refusal to participate. Table 1 presents the demographic information for participants who completed the study.

### 2.3. Treatment Materials and Procedures

The CST treatment protocol, adopted from the Chinese version of the CST manual translated by Wong [21], is based on the original version written by Spector et al. [13] (see Table 2 for the themes of each session). The protocol consisted of fourteen 45-minute group sessions, with at least two sessions per week in a group size of four to five. Every session began with reality orientation and snack time, followed by a 20/25-minute activity with themes such as physical games, food, and childhood. In the last five minutes of the session, the facilitator summarized the activities conducted and gave the participants a hint about the theme of the upcoming session.

The materials used in the current study were based on those used in conventional group CST with some minor modifications. Photos/pictures were displayed in digital form on tablets (iPads) so they could be enlarged for clearer inspection. Videos were presented on large display monitors. Real objects such as toys or snacks (in Session 3: childhood and Session 4: food), dried herbs/flowers for making scented sachets in Session 8 (creativity), and objects belonging to different categories for sorting in Session 9 (object categorization) were utilized. All therapy sessions were led by a primary facilitator who had completed CST training and a therapy assistant, a staff member from the residential care home, to support the participants. All therapy sessions were delivered identically across groups.

Treatment delivery was monitored by a qualified CST trainer (W.W.) to ensure treatment fidelity.

### 2.4. Outcome Measures on Cognition

The primary outcome was the Hong Kong version of the Montreal Cognitive Assessment for the Visually Impaired (HKMoCA-VI) [22], an assessment of global cognition for individuals with visual impairment. It excludes all visual elements and covers different cognitive domains, including orientation, attention, abstraction, language, and delayed memory. To reduce the practice effect in the delayed memory sub-test potentially induced by multiple testing, the target items in each testing point were quasi-randomly drawn from the list of items that served as multiple choice cueing in the sub-test. Secondary outcome measures on different cognitive domains included: (a) digit span forward and backward to measure verbal short-term and working memory, respectively, (b) subtests of the Test of Everyday Attention [23], including elevator counting (total score = 7) and elevator counting with distraction (total score = 10) to measure sustained auditory attention and selective attention, respectively, and (c) category fluency of animal and transportation to assess semantic memory and executive functions. The number of correct items produced within one minute represented their performance in the task.

### 2.5. Outcome Measures on Language and Communication

A series of tasks assessing language and communication served as secondary outcomes, including (a) a synonym judgment test [24] to assess verbal comprehension at a word level. On each trial, a pair of words were presented auditorily and subjects had to decide if the pair of words were similar in meaning; (b) a procedural description of making a ham-and-egg sandwich to measure the number of main concepts produced based on the normative performance collected in the local population [25]; (c) the Cantonese version of the Amsterdam–Nijmegan Everyday Language Test (CANELT) [26], which portrays 20 real-life situations encompassing different communicative functions, was conducted to evaluate functional communication quantitively. Since five of the scenarios involved the presentation of real objects, they were removed during test administration to avoid potential misunderstanding. Scoring was based on the number of main concepts produced in each scenario; (d) Holden Communication Scale [27] was used to evaluate the participants’ communication and social behavior from a caregiver’s perspective. It was administered once before and after the intervention. Ratings were given by the formal carers in the residential home.

The above-mentioned tasks, except the Holden Communication Scale, were administered three times, 15 days before and after treatment, respectively, with each session at least three to four days apart to reduce practice effect.

Measures of the applicability of CST, including attendance and adherence to the therapy protocol, were collected by the CST administrators.

### 2.6. Statistical Analysis

All the data were collected and scored by two authors, T.H.T. and L.C.L. Nonparametric statistical comparison, the Tau-U test, was employed for all outcomes except the Holden Communication Scale. Raw scores were used in all measures. Tau-U statistics allow the correction of any positive baseline trend (i.e., when the baseline slope was 0.33 or above) and the demonstration of treatment effect in both single-case and small-group samples via a test of significance [28]. Tau-U and the *p*-value of each participant in each outcome measure were computed. A combined Tau-U value of participants was then calculated for each measure to examine the overall group performance.

## 3. Results

Descriptive summaries of each of the cognitive/language measures taken pre-/post-therapy are given in Table 3, while the raw scores obtained in all the outcome measures can be accessed via the open-access repository “https://osf.io/d4hme/ (accessed on 5 December 2025)”.

Table 4 summarizes the group-level comparisons of the outcome measures between the pre- and post-treatment examinations. Tau-U comparisons for each participant before vs. after CST are given in Appendix A. There is a significant improvement in MOCA-VI, the primary outcome measure. Moreover, significant gains in various secondary outcomes, including digit span forward and backward and verbal fluency tasks, are observed. Similarly, the outcomes on language and communication, including synonym judgment and CANELT, also demonstrate significant improvement after CST. We did not perform any statistical tests to compare the scores on the Holden Communication Scale before/after therapy. However, there is a decrease in scale ratings from 8.43 to 7.71 after therapy, indicating improvement in communicative functions.

Based on the facilitators’ report, the treatment adhered well to the protocol. All the participants finished CST. Therapy attendance exceeded 85% among all the participants.

## 4. Discussion

The present pilot study was the first attempt to investigate the feasibility and preliminary clinical outcomes of conventional CST in elderly individuals with concurrent dementia and visual impairment. Seven PwDVI completed both assessment and treatment in the conventional CST. Their performance on global and specific cognitive domains was evaluated and compared before and after therapy. The results of the current pilot study seem to support that conventional CST is beneficial for PwDVI as no deterioration in cognitive and language abilities was observed after therapy. The participants adhered to the therapy protocol well. A comparison at the group level before and after therapy revealed significant gains in global cognition, verbal short-term and working memory, executive functions, functional communication, and word comprehension. This study has enriched the body of CST research by providing preliminary evidence on PwDVI, investigating the treatment effects on both general cognition and its subcomponents. In line with previous findings [14,29,30], the participants in the conventional CST condition demonstrated significant overall cognitive improvement, as evidenced by the average HKMoCA-VI score. Such results further support CST as a promising treatment not only for people with dementia but also for PwDVI.

The positive gains in different cognitive components could be attributed to the design and nature of the themed sessions of CST. Generally speaking, in each session, participants are engaged in activities involving multisensory and multi-cognitive stimulation. The improvements in executive function could be related to the divergent thinking and problem-solving activities used in different sessions (e.g., in every session, participants are encouraged to produce names belonging to different categories at the beginning as a warm-up activity and in session 4 ‘food’, in which they are asked to design a menu for a festive gathering. Additionally, the improvement in short-term memory is encouraging as memory decline is often reported in PwD [31]. Improvements in short-term and working memory may be accounted for by the active use of memory across different activities, such as the recruitment of working memory in calculation (as in Session 11 ‘using money’ and Session 12 ‘number games’), and by the need for verbal working memory during discussion with other members. On the other hand, no significant changes were obtained in all the attention measures (i.e., TEA EC and TEA ECD). Such results are consistent with the existing findings in which no improvement in attention was observed after treatment [32]. However, it should be noted that the attention measures used in this study were based on the auditory modality only, whereas other modality measures, such as visual (i.e., the trail-making test), were used in previous studies.

The improvements in language and communication have provided an objective evaluation of how language functions might improve through CST. Consistent with a previous study by Spector et al. [13], the participants showed a trend of improvement in social communication as judged by their caregivers using the Holden Communication Scale. The improvement in language comprehension and communication might be attributed to the nature of CST. The CST protocol includes several themes related to daily life, such as childhood, food, and using money. In each themed session, the participants were actively engaged in verbal interactions with the CST facilitators and other participating PwD. They were encouraged to share their opinions and experiences, and they also listened to the guiding questions and instructions from the facilitators and to the comments/personal sharing of other participating PwD. Spector et al. [33] suggested that the essence of CST is to promote verbal communication through implicit learning. This may explain why the participants showed improvements in both comprehension and expression. Moreover, Spector et al. [13] suggested that the group setting of CST is usually novel to nursing home residents because there is no obligatory context for them to communicate given their routinized daily life. CST provides an opportunity for them to exercise their long-unused communication skills and therefore improve them.

## 5. Limitations and Suggestions for Future Studies

This study encountered several methodological limitations. Firstly, the small sample size reduced its statistical power and generalizability. It should be noted that not all the participants had a formal diagnosis of dementia, nor were their subtypes of dementia known. Moreover, some participants did not receive any education, making it difficult to compare their performance with norms for various tests for which education-referenced norms were not available. The assessment used in the current study may not be comprehensive enough to cover all the cognitive domains given the lack of culturally adapted tests for the visually impaired Chinese population. Hence, the current study does not allow for the comparison of recovery patterns among participants across different cognitive domains. Thus, given the heterogeneity of the sample and the limitations in the methodology, the current study could only provide preliminary findings on the effects of CST on PwDVI, and the results should be treated with caution. The efficacy of conventional CST should be further investigated in an assessor-blind RCT with a no-treatment control group. A more rigorous methodology, including a set of well-defined inclusion criteria, characterization of participants’ condition (including dementia subtypes), and a more comprehensive norm-referenced assessment of cognitive domains as outcome measures, would allow us to study the effects of CST across different dementia subtypes and their patterns of recovery. Secondly, the study period could be extended to examine maintenance effects. In addition, interviews with participants and facilitators after therapy may provide a clearer understanding of their experience with CST, and their feedback may further enhance its delivery to PwDVI. Last but not least, communication between PwDVI and their facilitators can also be examined, which may be useful to identify strategies to further enhance their experience and promote interactions during CST [34].

## 6. Conclusions

This study provided preliminary evidence for conventional CST in VI populations with dementia. The positive results suggest that conventional CST could be applied to PwDVI.

## Figures and Tables

**Table 1:** The demographic information of the participants.

Participant	Age (Years)	Gender	Formal Diagnosis of Dementia	Level of Visual Impairment	Education Level (Years)	Baseline MoCa-5 Score
1	90	F	Yes	Moderate	Nil	5
2	96	F	No	Moderate	Nil	6
3	93	F	No	Moderate	Nil	12
4	96	F	Yes	Moderate	Nil	3
5	90	M	Yes	Moderate	unknown	5.5
6	79	M	No	Moderate	Nil	16
7	86	M	No	Moderate	9	13

Note. F = female; M = male; MoCA-5 = the Montreal Cognitive Assessment 5-Minute Protocol.

**Table 2:** The themes and materials of the CST program.

Session	Theme	Types and Quantities of Stimuli Used	
		Magnified Photos	Videos
1	Physical games	NA	
2	Sound	1	7
3	Childhood	12	3
4	Food	5	4
5	Current affairs	0	0
6	Faces/scenes	16	0
7	Word association	9	0
8	Being creative	6	1
9	Categorizing objects	14	0
10	Orientation	8	0
11	Using money	14	0
12	Number games	18	0
13	Word games	0	0
14	Team competition	NA	

Note. NA = not applicable.

**Table 3:** Descriptive summary of the performance on cognitive measures.

HKMoCA-VI				Verbal Fluency (Animal)				Verbal Fluency (Transportation)				Verbal Fluency (Animal and Transportation)			
Pre		Post		Pre		Post		Pre		Post		Pre		Post	
M	(SD)	M	(SD)	M	(SD)	M	(SD)	M	(SD)	M	(SD)	M	(SD)	M	(SD)
7.24	(2.79)	9.00	(4.15)	5.81	(3.01)	7.33	(3.98)	2.43	(2.09)	4.48	(2.18)	8.14	(4.37)	11.52	(5.92)
Digit span forward				Digit span backward				TEA EC				TEA ECD			
Pre		Post		Pre		Post		Pre		Post		Pre		Post	
M	(SD)	M	(SD)	M	(SD)	M	(SD)	M	(SD)	M	(SD)	M	(SD)	M	(SD)
6.57	(1.12)	7.71	(1.23)	2.05	(1.66)	2.81	(1.57)	5.29	(1.90)	5.95	(0.97)	3.19	(2.71)	3.48	(3.25)

Note. M = mean; SD = standard deviation; HKMoCA-VI = Hong Kong version of the Montreal Cognitive Assessment for the Visually Impaired; TEA EC = Test of Everyday Attention—Elevator Counting Subtask; TEA ECD = Test of Everyday Attention—Elevator Counting with Distraction Subtask.

**Table 4:** Tau-U (effect size) of the averaged performance of different outcome measures between pre- and post-treatment evaluations.

Measures	Tau-U	*p*
Cognition		
HKMoCA-VI	0.38	0.047 *
Digit span (forward)	0.53	0.005 **
Digit span (backward)	0.39	0.039 *
TEA EC	0.14	0.457
TEA ECD	0.17	0.364
Verbal fluency (animal)	0.24	0.216
Verbal fluency (transportation)	0.65	0.0007 ***
Verbal fluency (summation of animal and transportation)	0.40	0.039 *
Language and communication		
CANELT	0.413	0.047 *
Synonym judgement	0.397	0.047 *
Procedural description	0.127	0.509

Note. * *p* < 0.05. ** *p* < 0.01. *** *p* < 0.001. CANELT = the Cantonese version of the Amsterdam–Nijmegan Everyday Language Test; HKMoCA-VI = Hong Kong version of the Montreal Cognitive Assessment for the Visually Impaired; TEA EC = Test of Everyday Attention—Elevator Counting Subtask; TEA ECD = Test of Everyday Attention—Elevator Counting with Distraction Subtask.

## Data Availability

The original data presented in the study are openly available in OSF at https://osf.io/d4hme/ (accessed on 5 December 2025).

## Abbreviations

The following abbreviations are used in this manuscript:

CANELT	Cantonese version of the Amsterdam–Nijmegan Everyday Language Test
CST	Cognitive Stimulation Therapy
HKMoCA-VI	Hong Kong version of the Montreal Cognitive Assessment for the Visually Impaired
MMSE	Mini Mental State Examination
MoCA-5	Montreal Cognitive Assessment 5-Minute Protocol
NICE	National Institute for Health and Care Excellence
PwD	People with Dementia
PwDVI	People with Dementia and Visual Impairment
TEA EC	Test of Everyday Attention—Elevator Counting Subtask
TEA ECD	Test of Everyday Attention—Elevator Counting with Distraction Subtask

## Appendix A

**Table A1:** Tau-U statistics comparing pre- and post-treatment performance on different outcome measures.

Cognitive Measures																
	HKMoCA-VI		Digit Span Forward		Digit Span Backward		TEA EC		TEA ECD		Verbal Fluency (Animal)		Verbal Fluency (Transportation)		Verbal Fluency (Summation of Animal and Transportation)	
Pt	Tau-U	*p*	Tau-U	*p*	Tau-U	*p*	Tau-U	*p*	Tau-U	*p*	Tau-U	*p*	Tau-U	*p*	Tau-U	*p*
1	−0.44	0.382	−0.33	0.512	−0.33	0.512	0.77	0.126	−0.33	0.512	0	1	−0.44	0.382	−0.11	0.827
2	0.77	0.126	0.66	0.190	0.44	0.382	−0.11	0.827	0	1	0.66	0.190	0.88	0.080	0.66	0.190
3	1	0.049 *	0.66	0.190	0.44	0.382	0.11	0.827	0.33	0.512	0.11	0.827	0.77	0.126	0.33	0.512
4	−0.33	0.512	0.33	0.512	0.33	0.512	−0.44	0.382	−0.33	0.512	−0.33	0.512	0.44	0.382	0.11	0.827
5	0.33	0.512	0.55	0.275	1	0.049 *	0.22	0.662	0.77	0.126	0.11	0.827	1	0.049 *	0.44	0.382
6	0.55	0.275	1	0.049 *	0.77	0.126	0.44	0.382	0.44	0.382	0.77	0.126	0.77	0.126	0.88	0.080
7	0.77	0.126	0.88	0.080	0.11	0.827	0	1	0.33	0.512	0.33	0.512	0.33	0.512	0.44	0.382
	CANELT				Procedural description				Synonym judgment							
Pt	Tau-U	*p*			Tau-U	*p*			Tau-U	*p*						
1	−0.556	0.275			−0.333	0.513			−0.667	0.190						
2	0.444	0.383			−0.556	0.275			0.778	0.127						
3	0.667	0.190			0.222	0.663			−0.111	0.827						
4	−0.333	0.513			0.333	0.513			0.556	0.275						
5	1	0.049 *			0.333	0.513			0.222	0.663						
6	0.667	0.191			−0.111	0.827			1	0.049 *						
7	1	0.049 *			1	0.049 *			1	0.049 *						

Note. * = *p* < 0.05; CANELT = the Cantonese version of the Amsterdam–Nijmegan Everyday Language Test; HKMoCA-VI = Hong Kong version of the Montreal Cognitive Assessment for the Visually Impaired; TEA EC = Test of Everyday Attention—Elevator Counting Subtask; TEA ECD = Test of Everyday Attention—Elevator Counting with Distraction Subtask; Pt = participant.
